# Changes in Microeukaryotic Communities in the Grand Canal of China in Response to Floods

**DOI:** 10.3390/ijerph192113948

**Published:** 2022-10-27

**Authors:** Wei Cai, Huiyu Li, Xin Wen, Huang Huang, Guwang Chen, Haomiao Cheng, Hainan Wu, Zhe Piao

**Affiliations:** 1 College of Environmental Science and Engineering, Yangzhou University, Huayang West Road #196, Yangzhou 225009, China; 2 Key Laboratory of Integrated Regulation and Resources Development of Shallow Lakes of Ministry of Education, Hohai University, Xikang Road #1, Nanjing 210098, China

**Keywords:** eukaryotic communities, the Grand Canal, flood influence, distribution and structure, assembly mechanics, functional analysis

## Abstract

Floods are frequent natural disasters and could have serious impacts on aquatic environments. Eukaryotic communities in artificial canals influenced by floods remain largely unexplored. This study investigated the spatiotemporal variabilities among eukaryotes in response to floods in the Grand Canal, China. Generally, 781,078 sequence reads were obtained from 18S rRNA gene sequencing, with 304,721 and 476,357 sequence reads detected before and after flooding, respectively. Sediment samples collected after the floods exhibited a higher degree of richness and biodiversity but lower evenness than those before the floods. The eukaryotic communities changed from Fungi-dominated before floods to Stramenopile-dominated after floods. The spatial turnover of various species was the main contributor to the longitudinal construction of eukaryotes both before the floods (*β*_SIM_ = 0.7054) and after the floods (*β*_SIM_ = 0.6858). Some eukaryotic groups responded strongly to floods and might pose unpredictable risks to human health and environmental health. For example, Pezizomycetes, Catenulida, Glomeromycetes, Ellipura, etc. disappeared after the floods. Conversely, *Lepocinclis*, Synurale, Hibberdiales, *Acineta*, Diptera, and Rhinosporidium were all frequently detected after the floods, but not prior to the floods. Functional analyses revealed amino acid metabolism, carbohydrate metabolism, translation, and energy metabolism as the main metabolic pathways, predicting great potential for these processes in the Grand Canal.

## 1. Introduction

The Grand Canal of China is the largest artificial river in the world, stretching 1797 km long. It was dug manually, and so is a product of human transformation of the natural environment [1]. In addition to the fundamental ecological functions involving natural rivers, the Grand Canal plays an important role in transportation and recreational activities, and these human activities could cause environmental stress to the canal ecosystem [2]. Microeukaryotes are fundamental for maintaining ecosystem functions in aquatic environments. They are useful indicators in evaluating local ecological health and accessing the stressful effects of anthropogenic pollution on aquatic ecosystems [3]. They also participate in many biogeochemical processes, such as organic matter storage and degradation, phosphorus (P) adsorption and transformation, nitrification, and denitrification [4]. Understanding the ecological characteristics of microeukaryotes is crucial for the preservation of the environment and the ecological management of the Grand Canal. However, knowledge regarding the structure and assembly patterns of eukaryotic communities in canals is still lacking.

Microeukaryotes are sensitive to environmental changes [5]. Significant shifts in the composition, abundance, and diversity of eukaryotic communities at both the spatial and temporal scales corresponding to environmental variations have been observed in river systems [6]. Huang et al. (2019) found that land use patterns and hydrological regimes affected the composition and diversity of riverine eukaryotic communities [7]. Hery et al. (2016) explored the spatial distribution of eukaryotic communities in mining environments and both eukaryotic community structure and composition responded to ecosystem characteristics [8]. Such research shows that differences in biological and physiochemical characteristics largely shape the assembly patterns of eukaryotic communities. With the increasing magnitude and frequency of extreme precipitation events as a result of global warming there has been more intense and frequent river flooding in recent years [9]. Changes in river discharge during flood seasons can restructure hydrologic and physiochemical flow networks, thus exerting significant consequences on microbial ecology by changing biological processes [10]. It has been reported that the flood event that occurred in the Yangtze River in 2010 shifted the picoplankton community composition in the East China Sea from being dominated by *Synechococcus* to being dominated by heterotrophic bacteria, including Actinobacteria, Flavobacteria, α-Proteobacteria, and γ-Proteobacteria [11]. Zoppini et al. found that an allochthonous input from an extreme river flood event represented a source of energy for microbial metabolism, with organic matter being processed at double the rate in waters at high flood impact levels [12]. Phytoplanktonic and heterotrophic microbial community profiles were both found to be affected by riverine inputs caused by the extreme flooding that occurred in the Po River in Italy in 2014 [12]. Several studies have been conducted to reveal the influence of extreme weather events on the microbial communities in natural rivers and oceans. However, the understanding of how canal ecosystems respond to flooding is still in its infancy. Canals suffer more from human interference compared with natural rivers, and this is mainly due to the interference of hydraulic and biochemical factors brought by long-term navigation [1,2]. Characterizing the dynamics of eukaryotic community structures and their assembly patterns across canal environments, and responses to flood events will help us predict ecological changes in the canal systems as well as provide a basis for future environmental protection and ecological restoration measures in canals.

In July 2020, a serious natural flooding event caused by extreme precipitation threatened 27 provinces across central and southern China. During this event, measurements indicated the highest average level of precipitation along the Yangtze River witnessed since 1961 [13]. After the Huaihe River No.1 Flood on 17 July, the Huai-Yangtze River Channel continued to discharge flood with a maximum flow of 7930 m^3^/s [14]. The Huai-Yangtze River Channel is the largest flood discharge channel in the lower reaches of the Huai River and could carry more than 70% of the flood from the upper and middle reaches of the Huai River into the Yangtze River [15]. During the flood seasons, the Grand Canal is continuously subjected to stress from the surrounding connected rivers, especially from the Yangtze River and the Huai-Yangtze River Channel [16]. With the discharge of floods in the Yangtze River and the Huaihe River, the hydraulic and biochemical conditions in the Grand Canal change rapidly, thereby influencing the local river ecology. In this paper, we present a spatiotemporal study of the eukaryotic communities existing in sediments of the Grand Canal in China both before and after this flood in 2020 by sequencing eukaryotic 18S rRNA gene amplicons with Illumina Miseq. This study aimed to address two questions: (i) What are the temporal and spatial patterns of eukaryotic communities in canal sediment in response to flood distribution? (ii) What are the mechanisms controlling the community diversity, succession, and biogeography of the eukaryotic communities before and after the floods? The results of this study will help resolve a research gap regarding eukaryotic ecologies and their responses to natural disasters in artificial canals and will also provide a basis for canal regulation due to the ecological implications of flooding, water conservation, and environmental management.

## 2. Materials and Methods

### 2.1. Study Area

**Figure 1:**
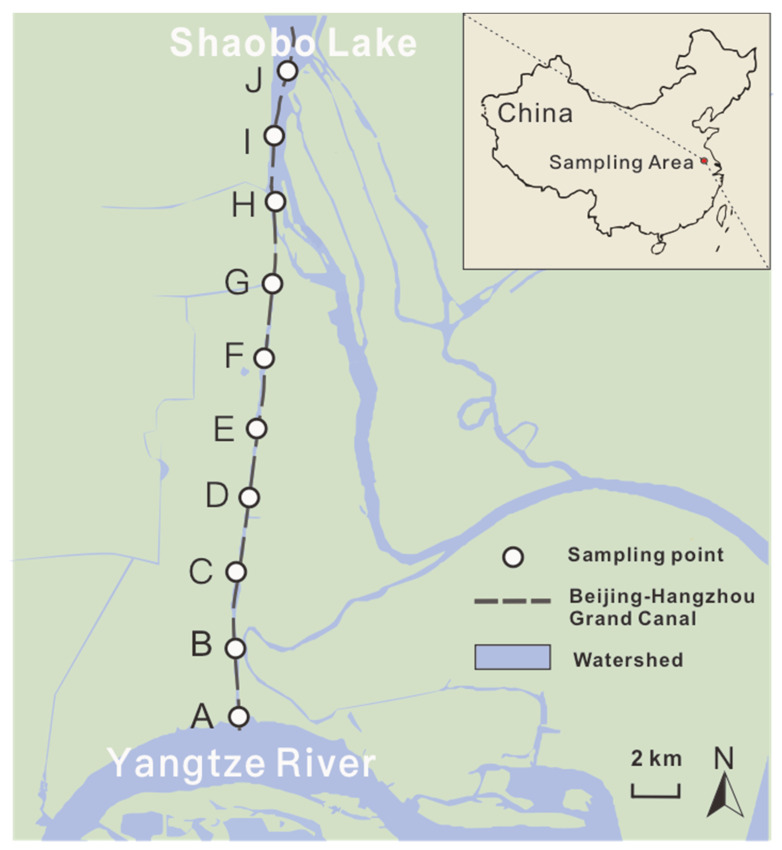
Locations of the sampling sites along the Grand Canal.

The Grand Canal starts from Hangzhou in South China and ends in Beijing in North China, passing through the Haihe River, Yellow River, Huaihe River, Yangtze River, and Qiantang River. The particular section of the Grand Canal that links to the Huai-Yangtze River Channel and Yangtze River was selected for study (119°28′–119°29′ E, 32°16′–32°29′ N) (Figure 1). The north part of this section connects to the Shaobo Lake, an important channel of the Huai-Yangtze River Channel, while the south part of the section reaches the Yangtze River. The study area was continuously subjected to stress from the Yangtze River and the Huai-Yangtze River Channel during the serious flooding in July 2020.

### 2.2. Sample Collection and Pretreatment

Water and sediment samples were collected from 10 sampling sites (Figure 1) along the above-chosen section of the Grand Canal linking the Huai-Yangtze River Channel and the Yangtze River in June (before the flood event) and September 2020 (after the flood event). Triplicate samples of river water from each site were sampled from a depth of 1 m below the water surface, mixed and gathered using 4-L polyethylene bottles, transported to the lab, and then processed within 2 h. Surface sediments (0–5 cm depth below the sediment-water interface) were collected in triplicate using the Van Veen grab sampler at each site, and then mixed into one homogenized composite sample. Small subsamples (about 1–2 g) of the homogenized sediments were packed into a sterile 2 mL centrifuge tube for subsequent DNA extraction and stored on ice until frozen at −80 °C.

### 2.3. Physiochemical Properties Analysis

Physiochemical properties of water samples were analyzed after being filtered through 0.45 μm filters (Millipore, Burlington, MA, USA). Water temperature (T), dissolved oxygen concentration (DO), pH and conductivity (Cond) were measured at the time of collection using a Horiba U-10 water quality checker. Concentrations of total nitrogen (TN), total phosphorus (TP), COD_Mn_ were determined based on previous studies [17]. Three replicates were performed for the analysis of physiochemical properties.

### 2.4. DNA Extraction and PCR Amplification

The DNA extraction and PCR amplification were performed using the primer sets TAReuk454FWD1 (5′-CCAGCASCYGCGGTAATTCC-3′) and TAReukREV3 (5′-ACTTTCGTTCTTGATYRA-3′), targeting the eukaryotic V4 region of the 18S rRNA genes [18]. Sequencing was then performed using Illumina 2500 platform by Shanghai Biozeron Biotechnology Co., Ltd. (Shanghai, China). The bioinformatics analysis was conducted according to the procedure described previously [19]. Raw sequencing data used for the analyses in this manuscript are available from the National Center for Biotechnology Information (NCBI) Sequence Read Archive with Accession No. PRJNA856781.

### 2.5. Statistical Analysis

All data analyses were conducted using R software (v.3.5.1, http://www.r-project.org). The package “vegan” was used to compare the diversity, richness, and evenness of eukaryotic communities based on the OTU. Variations in the structure and composition of eukaryotic communities among samples were visualized using principal component analysis (PCA) and principal coordinate analysis (PCoA) based on the distance matrix of normalized OTU abundances. Venn diagrams were used to visually depict taxa overlap in canal sediment before and after the floods at different category levels. PICRUSt2 (phylogenetic investigation of communities by reconstruction of unobserved states) analysis was used to determine the predicted Kyoto Encyclopedia of Gene and Genomes (KEGG) categories from the gene contents and abundances among eukaryotic community data [20].

## 3. Results

### 3.1. Physiochemical Properties

The main physicochemical properties of the water samples collected from different sites are listed in Table S1. The water temperature ranged between 18.8 °C–19.5 °C before the floods and 25.1 °C–25.7 °C after the floods. High nutrient levels were observed based on the average values of TN (4.76 mg/L before the floods and 4.95 mg/L after the floods) and TP (1.02 mg/L before floods and 0.95 mg/L after the floods). The average pH and DO values were both higher before the floods than after the floods, while those of Cond and COD_Mn_ exhibited the opposite trend.

### 3.2. Species Richness, Evenness, and Diversity

Table 1 shows the statistics of the data for sequence reads and gene alpha diversity indices among the samples. Generally, a total of 781,078 sequence reads were obtained among 20 samples by using 18S rRNA gene sequencing, with 304,721 and 476,357 sequence reads detected before and after the floods, respectively. The sequences were assigned to 7152 (before the floods) and 9474 OTUs (after the floods) with a 97% sequence identity threshold. Substantial fluctuations were observed in the numbers of sequence reads and OTUs among all the sampling, ranging from 5149 to 80,321 and 364 to 1198, respectively. Overall, the sediment samples collected after the floods exhibited higher levels of richness and biodiversity but lower evenness than those collected before the floods. The Chao1 and ACE estimators showed higher average values after the floods (1209.4 and 1224.6, respectively) than those before the floods (847.8 and 855.4, respectively). The average Simpson index of diversity decreased from 0.1014 before the floods to 0.0916 after the floods, and the Chao1 index increased from 717.0 before the floods to 949.5 after the floods. Pielou’s species evenness index was 0.63 on average before the floods and decreased to 0.56 after the floods.

### 3.3. Composition and Distribution Characteristics of Eukaryotic Communities

**Figure 2:**
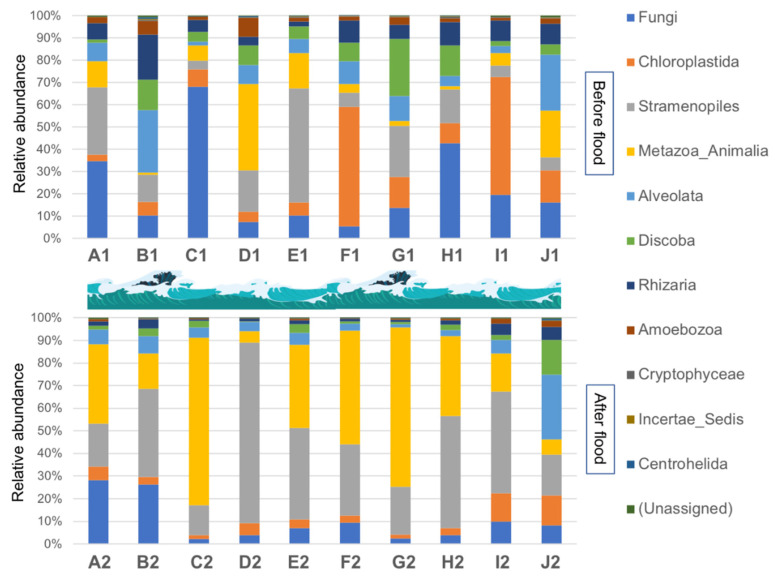
Relative abundance of dominant kingdoms of the eukaryotic communities.

**Figure 3:**
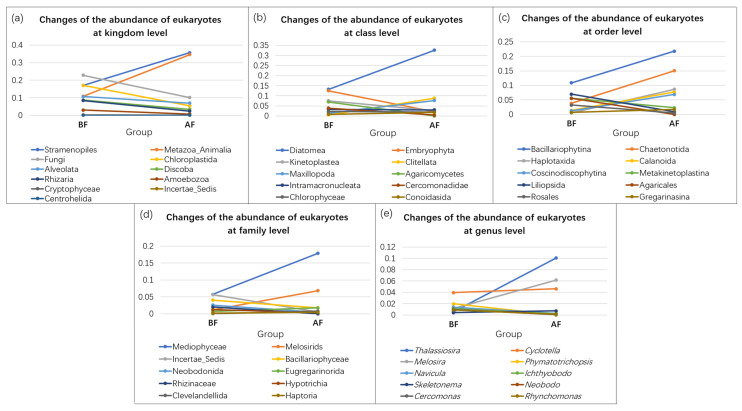
Changes in the relative abundance of eukaryotes before and after the floods at the kingdom level (**a**), class level (**b**), order level (**c**), family level (**d**), and genus level (**e**).

Among the 20 samples, a total of 11 kingdoms, 89 classes, 135 orders, 82 families, and 223 genera were identified and assigned. The composition and distribution characteristics of eukaryotic communities at the kingdom level are shown in Figure 2. Generally, Stramenopiles, Metazoa_Animalia, and Fungi were the top three most abundant groups, comprising 26.38%, 22.73%, and 16.50% of the total average of 20 samples. Among the 10 samples collected before the floods, the majority of the OTUs were clustered into eight high-level taxonomic groups, including Fungi (22.86%), Chloroplastida (17.12%), Stramenopiles (17.06%), Metazoa_Animalia (10.85%), Alveolat (10.77%), Discoba (8.74%), Rhizaria (8.49%), and Amoebozoa (3.07%). Three minor kingdoms (Cryptophyceae, Incertae_Sedis, and Centrohelida.) jointly represented less than 1% of the total sequences. Among the 10 samples collected after the floods, Stramenopiles (35.70%), Metazoa_Animalia (34.62%), Fungi (10.14%), Alveolat (6.98%), Chloroplastida (5.37%), Discoba (3.43%), and Rhizaria (2.46%) comprised the high-level taxonomic groups. Other groups (Amoebozoa, Cryptophyceae, Incertae_Sedis, and Centrohelida) occupied only a small proportion (1.14%) of the total sequences. Metazoa_Animalia and Alveolata showed higher relative abundances among samples after the floods than before the floods, while the abundances of Fungi, Chloroplastida, Stramenopiles, Metazoa_Animalia, Alveolata, Discoba, Rhizaria, Amoebozoa, Crytophyceae, Incertae_sedis, and Centrohelida all decreased after floods (Figure 3a).

At the class level, the most abundant group was Diatomea both before and after the floods. Its average abundance increased from 13.26% before the floods to 32.61% after the floods (Figure 3b). Embryophyta and Kinetoplastea followed by Diatomea were the top three abundant groups before the floods, while Clitellata and Maxillopoda were identified as the second and third most abundant groups after the floods. The dominant eukaryotic orders were Bacillariophytina (10.87%), Liliopsida (6.98%), and Metakinetoplastina (5.58%) before the floods. After the floods, Bacillariophytin was also the most abundant order, accounting for 21.79% of the total sequences, followed by Chaetonotida (15.03%), and Haplotaxida (8.62%). The relative abundance of Bacillariophytin, Chaetonotida, Haplotaxida, Calanoida, and Coscinodiscophytina all showed an obvious increase after the floods (Figure 3c). Mediophyceae, Incertae_Sedis, and Bacillariophyceae were the top three most abundant families before the floods, accounting for 5.74%, 5.71%, and 4.02% of the total sequences on average. After the floods, the relative abundance of Mediophycea increased to 17.87%, thereby remaining the most abundant group (Figure 3d). The abundance of Incertae_Sedis decreased to 0.57%, conferring the second highest abundance of Melosirids (6.84%). Bacillariophyceae was the third most abundant group, but only comprised 1.78% of the total. The top five dominant genera from sediment samples collected before floods were *Cyclotella* (3.94%), *Phymatotrichopsis* (1.98%), *Navicula* (1.39%), *Melosira* (1.16%), and *Ichthyobodo* (1.13%). The abundance of *Thalassiosira* and *Melosir* both showed obvious increases after the floods (Figure 3e). Only three genera, including *Thalassiosira* (10.04%), *Melosira* (6.15%), and *Cyclotella* (4.62%) each accounted for more than 1% of the abundance in the sediments after the floods. 

### 3.4. Multivariate Analysis of Eukaryotic Communities

#### 3.4.1. PCoA Analysis and Venn Analysis

**Figure 4:**
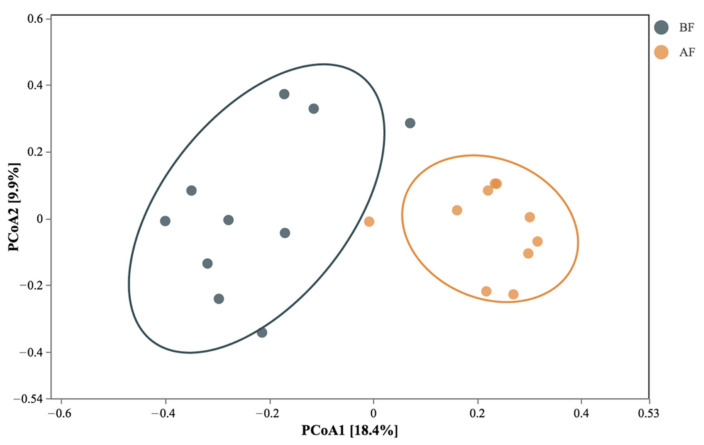
Comparison of eukaryotic communities before and after the flood using principal coordinates analysis (PCoA) (BF represents samples collected before floods and AF represents samples collected after floods).

**Figure 5:**
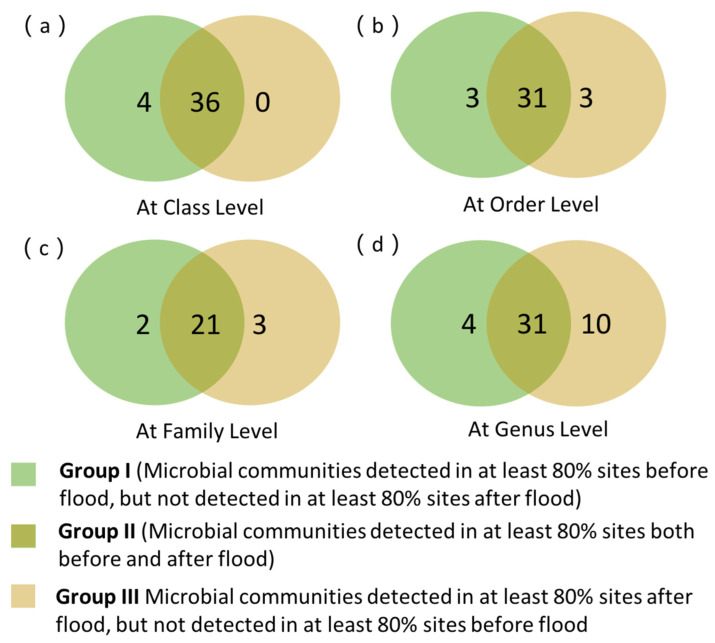
Venn diagram demonstrating shared and unique eukaryotic taxa at class, order, family, and genus level in samples collected before and after the floods. Detailed data of eukaryotic taxa are shown in Table S2.

The bioplot of the PCoA ordination based on Bray-Curtis dissimilarity matrices of OUT at a 97% cutoff was performed to reveal the community-level differences of the eukaryotes before and after floods. As shown in Figure 4, the first and second principal components explained 18.4% and 9.9% of the variation, respectively. Our results indicated that the eukaryotic communities in the canal sediment before and after the floods could be clearly separated. A Venn diagram illustrating the numbers of shared and unique eukaryotic taxa at the class, order, family, and genus levels in the samples collected before and after the floods is shown in Figure 5. Group I includes the eukaryotic communities that were detected in at least 80% of the sampled sites before the floods, but not detected in at least 80% of the sites after the floods. Group II includes the eukaryotic communities that were detected in at least 80% of the sampled sites both before and after the floods. Group III includes taxa detected in at least 80% of the sampled sites after the floods but not detected in at least 80% of the sites before the floods. Most of the eukaryotic communities belonged to Group II, indicating a great level of similarity in the eukaryotic communities before and after the floods. At the kingdom level, all kingdoms were assigned to Group II, which means that the eukaryotic communities detected before and after the floods were identical at this category level. At the class level, 36 eukaryotic classes were classified into Group II as frequently occurring communities both before and after the floods, while 4 classes, including Pezizomycetes, Catenulida, Glomeromycetes, and Ellipura appeared in Group I as frequently occurring communities before the floods. At the order level, 3 orders (Pezizales, Tylenchida, and Collembola) were frequently detected before floods, while the remaining 3 orders (Diptera, Synurales, and Hibberdiales) were commonly found after floods. The results also showed that few groups, including 2 families (Rhizinaceae and Stenostomidae) and 4 genera *(Phymatotrichopsis, Gonostomum, Hanseniaspora,* and *Angulamoeba*) belonged to Group I. A total of 3 families (Pichiaceae, Oligotrichia, and Armophorida) and 10 genera *Acineta, Halteria, Epiphyllum, Brachonella, Paulinella, Mallomonas, Lepocinclis, Katablepharis, Pichia,* and *Rhinosporidium* were categorized into Group III.

#### 3.4.2. Functional Analysis from PICRUSt2

**Figure 6:**
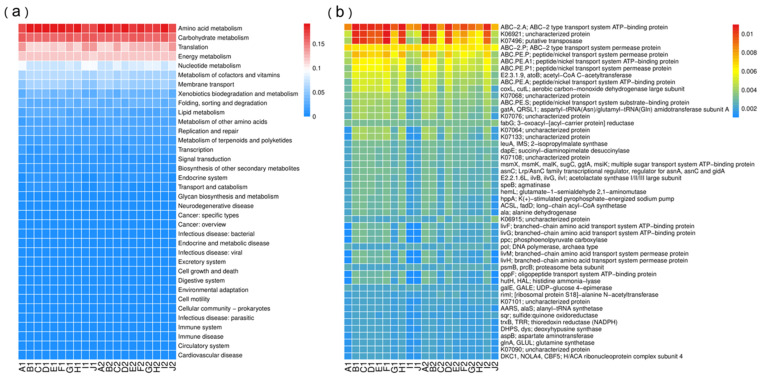
Heatmaps based on the relative abundance of the predicted metagenomes of KEGG genes (**a**) and metabolic pathways (**b**).

To elucidate the potential functions of eukaryotic communities in the Grand Canal, the KEGG Orthology (KO) database was utilized to classify the metabolic pathways into 6 categories based on PICRUSt2 metagenomes, including metabolism (related genes comprising 71.48% of the total genes on average), genetic information processing (19.12%), environmental information processing (6.76%), cellular processes (0.76%), organismal systems (1.04%), and human diseases (0.84%). The heatmaps depict the relative abundance of the top 50 predicted metagenomes and the main specified KEGG pathways among the samples in Figure 6a,b, respectively. ABC-2.A, the ATP-binding protein component of the ABC-2 type transport system, is the most abundant functional gene, comprising 0.96% of the total genes on average. Among 36 metabolic pathways, amino acid metabolism, carbohydrate metabolism, translation, and energy metabolism were the most abundant groups, with each being relatively over 1% abundant in all samples. Functional genes associated with amino acid metabolism and carbohydrate metabolism were more abundant before the floods, while those related to translation and energy metabolism showed higher abundance after the floods.

### 3.5. β-Diversity Measurement

**Figure 7:**
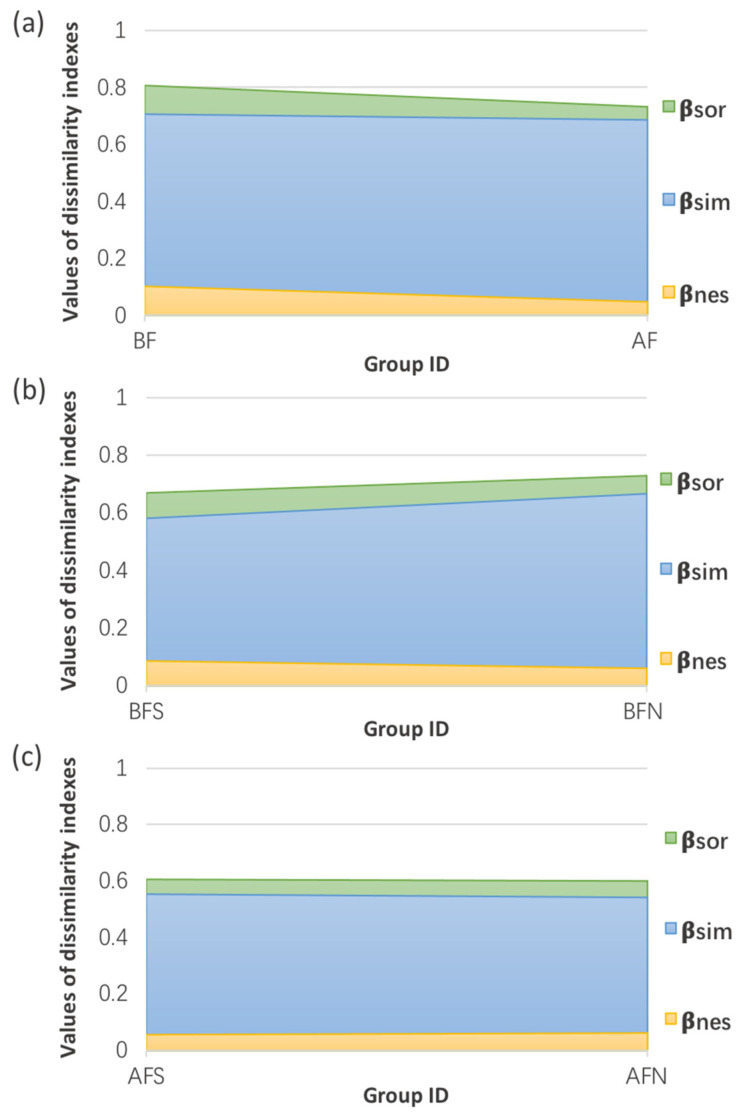
*β*-diversity pattern indexes of eukaryotic communities (**a**) before and after the floods (BF represents samples collected before the floods; AF represents samples collected after the floods); (**b**) in the southern and northern parts of the sampling sites before the floods (BFS represents samples collected in the southern part of the sampling sites before the floods; BFN represents samples collected in the northern part of the sampling sites before the floods); (**c**) in the southern and northern parts of the sampling sites after the floods (AFS represents samples collected in the southern part of the sampling sites after the floods; AFN represents samples collected in the northern part of the sampling sites after the floods).

Figure 7 shows the dissimilarities in the spatial turnover and nestedness pattern indexes of eukaryotic communities according to Baselga’s community assembly method [21]. Detailed calculation formulas are described in the supporting information. Species nestedness and spatial turnover represent two phenomena typically used to reflect the *β*-diversity patterns (variation of the species composition of assemblages) of eukaryotic communities. As a result of any mechanism that encourages the orderly disaggregation of assemblages, nestedness develops when the biotas at certain locations with fewer species are subsets of the biotas at sites with more species. In contrast to nestedness, spatial turnover denotes the substitution of one species for another as a result of environmental sorting or geographic and historical limitations. The Sørensen dissimilarity index (*β*_SOR_), Simpson dissimilarity index (*β*_SIM_), and nestedness-resultant dissimilarity index (*β*_NES_) were used to describe the similarities in the eukaryotes among the sampled sites. *β*_SIM_ depicts the spatial turnover of species among multipoint eukaryotes without the impact of richness gradients; *β*_SOR_ is associated with the percentage of different species shared among various communities; *β*_NES_ indicates the species nestedness among multipoint biotas.

The detailed data of the dissimilarity indexes are summarized in Table S3. By comparing the dissimilarity indices of eukaryotic communities among samples before the floods, when sample classification took the flood occurrence as the dividing line, the *β*_SIM_ values (0.7054) were higher than the *β*_NES_ values (0.1027). A similar trend was observed for the samples collected after the floods, where the *β*_SIM_ values was 0.6858, which was much higher than the *β*_NES_ values (0.0459). The *β*_SOR_ values was 0.8082 before the floods and decreased to 0.7318 after the floods. *β*_SIM_ decreased when the samples were divided into four groups based on both time and space, including the southern region before the floods (BFS), the northern region before the floods (BFN), the southern region after the floods (AFS), and the northern region after the floods (AFS). However, *β*_SIM_ still exhibited higher values than *β*_NES_ in the four groups mentioned above. The *β*_SOR_ values in the BFS, BFN, AFS, and AFN groups were calculated as 0.6673, 0.7275, and 0.6002, respectively, which were all lower than those determined for the BF and AF groups. The dissimilarity indices of samples did not have a unified change trend in the southern and northern regions of the canal. The *β*_SIM_ value was higher in the BFN group than the BFS group and was also higher in the AFN group compared to the AFS group.

## 4. Discussion

The Grand Canal of China plays a great role in transportation, irrigation, flood diversion, drainage, water supply, and water transfer. Nowadays, the state of climate change is becoming more and more complex, and substantially affects the ecology of the Grand Canal [1]. Eukaryotic communities contribute to a vital ecological function by participating in many biological processes in river systems. However, despite this, they have received little research attention in canals. In this study, the influence of flood events on the distribution and assembly patterns of eukaryotic communities in the Grand Canal was investigated.

The physicochemical properties of sampled water showed substantial changes when compared between before and after the floods, and this was mainly reflected by the pH value and the concentrations of DO and COD_Mn_. Rapid changes in river runoff caused by floods can scour the non-point pollution source and the internal source in the river [22]. The increase in the average COD_Mn_ concentration after floods indicates that large amounts of organic pollutants may have been carried or washed by the floods, thereby deteriorating the water quality of the Grand Canal. Simultaneously, organic pollutants can consume a great amount of DO in the decomposition process and produce some acidic substances, probably leading to the reduced pH and DO concentration observed in the canal water. Previous studies have demonstrated that the hydrodynamic and physicochemical conditions of a water body can exert a serious impact on the microbial ecology in aquatic environments [23,24]. The causal distinction of the processes promoting biodiversity may be explained by the entire development of the pH, DO, nutrient content, and electron acceptors during flood events [25,26]. Reis et al. identified the spatial heterogeneity and hydrological fluctuations caused by the flood pulse as the main factors shaping the bacterioplankton community composition in an Amazon floodplain system [27]. The spatiotemporal variations in the assembly patterns of the eukaryotic communities comprising the Grand Canal are thought to result from these developments during the floods. Our results showed that the flood distribution could lead to a higher richness, higher diversity, and lower evenness of the eukaryotic communities residing in canal sediments after the floods. It is not surprising that this trend could occur due to the improvement of river connectivity in transition and habitat homogeneity after the floods. The *β*-diversity measurements revealed the internal mechanisms underlying community diversity. Generally, the longitudinal development of the eukaryotic communities in the sediment along the Grand Canal is a comprehensive result of both the species’ spatial turnover and their nestedness. The spatial turnover of species was the main contributor to the longitudinal construction of the eukaryotic community structure in the Grand Canal, which was consistent before and after the floods. The nestedness of species assemblages signify the non-random species loss caused by any factor that encourages the disaggregation of assemblages, and spatial turnover denotes the replacement of some species and acts as a result of environmental categorization or spatial and historical limitation [21]. The studied eukaryotic communities exhibited varied dissimilarity patterns before and after the floods, whereby the estimated overall *β*-diversity was lower for the samples collected after the floods. When considering species turnover and nestedness components separately, each contributed less to the longitudinal construction of eukaryotes after the floods than before the floods.

The distribution patterns of the dominant taxonomical groups varied before and after the floods. Generally, Stramenopiles, Metazoa_Animalia, and Fungi were determined as the most abundant groups in the Grand Canal in this study. This is similar to previous findings where they have commonly been found as the main eukaryotic groups present in the surface water body [28,29]. We also found that the eukaryotic communities changed from Fungi-dominated to Stramenopile-dominated. Fungi were identified as one of the main components involved in the aquatic food chain and the degradation of refractory organic matter. The ecological characteristics of Fungi could also influence the composition and distribution of bacteria and protozoa in the aquatic environment [30,31]. The observed decrease in the abundance of Fungi in the canal sediments after floods also likely indicates a reduced ability in the degradation of refractory organic matter by Fungi. According to the phylogenetic analysis, the detected Stramenopiles comprised both photosynthetic and non-photosynthetic creatures (such as oomycetes and labyrinthulas). Photosynthetic Stramenopiles act as an important member in aquatic primary productivity and biomass production, owing to their secondary endosymbiotic chloroplasts [32]. Chrysophyceae has sometimes been found to be the dominant photosynthetic eukaryote in river systems in previous reports [33,34], but was not found to be particularly abundant in our study. Instead, Diatomea, which belongs to the Stramenopiles clade, occupied the highest proportion among various classes. Huang et al. (2019) investigated the spatiotemporal variations of riverine eukaryotic communities and found that different types of watersheds are characterized by different dominant groups. It was revealed that Animalia was the most abundant group in agricultural watershed samples, while Stramenopiles and Alveolata dominated the eukaryotes occupying urban and forest watershed samples [7]. Therefore, unlike the absolute dominance of Proteobacteria observed within bacterial communities in different kinds of freshwater areas [35,36], the dominant eukaryotic community varies among different aquatic environments under various physiochemical conditions. Under conditions involving increasingly disruptive environmental changes caused by climate change, it is likely that greater variations will occur in the assembly patterns of eukaryotic communities in the aquatic environment.

PICRUSt2 analysis revealed that different gene and pathway categories showed no obvious differences before and after floods, probably indicating that the floods had little impact on the functions of eukaryotes in the Grand Canal. Metabolism-related genes were determined as the most dominant functional group, mainly composed of amino acid metabolism, carbohydrate metabolism, and energy metabolism. Consequently, it can predict great potential in the amino acid metabolism, carbohydrate metabolism, and energy metabolism processes in this system. Although there were no glaring discrepancies in the functional properties of eukaryotes, comparisons of the unique and rare eukaryotic taxa before and after the floods (detailed seen in Table S2) revealed a batch of eukaryotes with what we denote as “flood response” organisms. Pezizomycetes, Catenulida, Glomeromycetes, Ellipura, Pezizales, Tylenchida, Rhizinaceae, Stenostomidae, Collembola, *Phymatotrichopsis, Gonostomum, Hanseniaspora,* and *Angulamoeba* all exhibited a strong response to the flood event, as they all disappeared after the floods. Pezizomycetes, mainly represented by *Phymatotrichopsis,* were found to have frequently occurred in the canal sediments obtained before floods, with an average high relative abundance of 2.0%, but could not be detected after floods. Pezizomycetes are commonly found around the world, but their representative constituent taxonomic groups are unevenly distributed [37]. Pezizaceae (belonging to Pezizomycetes) has been reported to show a particularly high diversity in temperate regions [38]. Glomeromycetes were another typical group observed in Fungi that showed a unique appearance only before floods, but with a relatively lower average abundance of 0.12%. It is generally found in soil environments and has close symbiotic relationships with its plant hosts and can assist plants in capturing nutrients such as nitrogen, sulfur, and phosphorous [39]. Flood events might destroy their hosts and themselves, and this may explain the disappearance of Glomeromycetes after floods. Catenulida and Ellipura (belonging to Metazoa_Animalia) were commonly found in freshwater habitats [40] and were also found to frequently occur before the floods, with an abundance of 0.37% and 0.08%, respectively. Catenulida is a group of small worms comprising about 100 species in the world, most of which live in freshwater habitats such as rivers, mires, ponds, and moist terrestrial areas [41]. The absence of Pezizomycetes, Glomeromycetes, Catenulida, Ellipura, and other taxa after the floods probably indicates their poor adaptability under the fluctuating environment affected by drastic alterations in physiochemical and hydrological conditions. Conversely, *Lepocinclis*, Synurale, Hibberdiales, *Acineta*, Diptera, and *Rhinosporidium* comprised the few typical taxa that were detectable only after floods, and these may pose risks to human health and the environmental health of the canal system. The sudden appearance of these taxa after floods might be due to their introduction from the upstream area with a certain randomness. *Lepocinclis* is a common phytoplankton usually found in lakes and small still water bodies, and it turns the water green when it proliferates in large numbers [42]. Synurale and Hibberdiales both belong to Chrysophyceae, an important part of the freshwater algae. They typically reside in puddles, rice fields, ponds, and swamps [43]. When *Lepocincli* proliferates in large numbers in aquatic environments, the water area could turn green. When Synurale and Hibberdiale reproduce in large numbers in aquatic environments, green water and a fishy odor can be detected [44]. Therefore, the occurrence of *Lepocincli*, Synurale, and Hibberdiale after floods could pose potential environmental risks to the ecological environment in these rivers. In addition to such environmental risks, potential hidden dangers to organisms and human health also come with the occurrence of flood events. The floods brought new taxa that were never observed before the floods, including *Acineta, Diptera, Rhinosporidium,* and so on. *Acineta* is frequently detected in sediment collected after floods, and it is a fixed parasite that often attaches to the bodily surface, gills, and appendages of shrimp, crabs, shrimp eggs, and larvae. *Acineta* can slow and distort the movements of shrimp and crabs, make it difficult for them to eat, induce gill tissue degeneration, cause occlusion, block hypoxia, and finally make them emaciated and exhausted [45]. Diptera is the fourth largest order of Insecta after Coleoptera, Lepidoptera, and Membranoptera, and is commonly found around the world. It is extremely adaptable to various harsh environments. Some members of Diptera are vectors for transmitting diseases between people, animals, and plants [46,47]. *Rhinosporidium* is another eukaryotic pathogen that compromises human health. It is sometimes detected in lakes, ponds, and soil. Animals such as horses and cattle are the storage hosts of this microorganism, which can be transmitted to humans. *Rhinosporidiu seeberi* could infect both human and animal mucosal epithelium and cause Rhinosporidiosis through contact with contaminated water or soil [48]. Although we detected only a low abundance (<1% on average) of such “dangerous” taxa (*Lepocinclis*, Synurale, Hibberdiales, *Acineta*, Diptera, and *Rhinosporidium)* among the samples collected after floods, they could still pose an unpredictable risk to the river ecology and human health, as long-term species reproduction and growth can occur. Therefore, the occurrence of these taxa after floods might serve as a warning to pay further attention to the rivers and thereby potential increased environmental risks.

Previous research has shown that physicochemical properties and hydrological regime parameters govern the spatiotemporal variations of eukaryotic communities [5,8]. Under normal conditions, the canal environment is characterized by low velocity, low discharge, and a relatively stable physicochemical environment. But following flooding, the hydrological and physicochemical conditions change dramatically [49]. Some eukaryotes move to new habitats with the flow, which might explain the appearance of new species detected after floods. Increases in water velocity and flow discharge reduce the stability of the canal environment. A high load of sediment and other suspended solids were discharged into the Grand Canal, accompanied by industry and agricultural materials containing heavy metals, pesticides, and other pollutants. When these pollutants enter bodies of water, they pollute the drinking water source. The area of the Grand Canal investigated in this study is also a section of the East Line of the South-to-North Water Transfer Project, where the health of river ecology is of significant importance. The composition and diversity patterns of eukaryotic communities undergo spatiotemporal changes with the evolution of their habitat rivers when floods intensify or change this trend to some extent. Previous studies have also claimed that any change in species composition is driven by stochasticity in species birth and death rates and/or the deterministic fitness differences between taxonomic groups after perturbations [50]. As a result of the abiotic effects, a different selection of processes contributed to the assembly patterns of eukaryotic taxon assemblages. Flood disturbances could lead to considerable changes in various environmental properties, which might alter the interspecies interactions (e.g., predation, competition, and mutualisms) and thus cause variations in eukaryotic taxon assemblages.

Floods have now become one of the most frequent natural disasters in many countries around the world [9]. Flooding events can exert serious impacts on canal ecological environments. Therefore, attention should be paid to the ecological restoration and management of canals as the environment after floods may be different. Our study provides a theoretical basis for water pollution control and ecological restoration in canals. To advance our understanding of the effects of floods on the canal ecology, more extensive investigations into the structure, composition, assembly patterns, and ecological functions of eukaryotic communities should be carried out along the Grand Canal and in other canals. Further studies on the influence of mechanics on eukaryotic species succession responding to flood events should also be further investigated through a combination of on-site tests and laboratory tests.

## 5. Conclusions

The Grand Canal of China is the largest artificial river in the world. In the summer of 2020, severe natural flooding caused by extreme precipitation threatened 27 provinces across central and southern China. Given the relevance of the ecological processes carried out in the Grand Canal and the vital role of eukaryotic communities in these processes, this study investigated the spatiotemporal variabilities among eukaryotic communities in response to this flood event in the Grand Canal. A total of 781,078 sequence reads were obtained through gene sequencing of the 18S rRNA, with 304,721 and 476,357 sequence reads detected before and after the floods, respectively. Generally, Stramenopiles, Metazoa_Animalia, and Fungi were the most abundant taxonomic groups detected in the Grand Canal. The longitudinal development of the eukaryotic communities inhabiting the sediment along the Grand Canal is a comprehensive result of both the species’ spatial turnover and nestedness. The spatial turnover of species was the main contributor to the longitudinal construction of eukaryotes both before the floods (*β*_SIM_ = 0.7054) and after the flooding (*β*_SIM_ = 0.6858). Samples collected after the floods exhibited higher degrees of richness and biodiversity but lower evenness than those collected before the floods. The eukaryotic communities changed from Fungi-dominated before the floods to Stramenopile-dominated after the floods. Functional analyses revealed amino acid metabolism, carbohydrate metabolism, translation, and energy metabolism as the main metabolic pathways, allowing for predicting the potential of these processes in the Grand Canal. Comparisons of the unique and rare eukaryotic taxa before and after the floods revealed a batch of eukaryotes with a “flood response”. Pezizomycetes, Catenulida, Glomeromycetes, Ellipura, and others appeared to have a strong response to floods, as they all disappeared after the floods. Conversely, *Lepocinclis*, Synurale, Hibberdiales, *Acineta*, Diptera, and *Rhinosporidium* were frequently detected after floods, but not prior to the flood, and these alterations in their abundances pose unpredictable risks to human health and the environmental health of the canal system. Our study provides a theoretical basis for water pollution control and ecological restoration in canals.

## Figures and Tables

**Table 1:** Sequencing statistics and alpha diversity indices of eukaryotic communities among samples.

Sample ID	Reads	OTUs	ACE	Chao1	Shannon	Simpson	Pielou
A1	72,281	1198	1366.2	1345.0	4.15	0.941	0.58
B1	44,532	828	926.7	961.4	5.02	0.983	0.75
C1	80,321	1122	1260.4	1262.7	2.90	0.732	0.41
D1	48,964	1070	1168.4	1180.5	4.30	0.896	0.62
E1	15,962	567	676.9	698.1	4.09	0.931	0.64
F1	5917	330	511.6	488.3	2.77	0.728	0.48
G1	7975	526	651.2	671.3	5.00	0.982	0.80
H1	11,450	560	705.2	691.7	4.49	0.955	0.71
I1	5149	364	477.9	472.2	3.56	0.882	0.60
J1	12,170	587	733.3	783.1	4.37	0.955	0.69
Mean	30,472	715	855.4	847.8	4.07	0.899	0.63
A2	25,226	798	990.6	1017.0	3.67	0.882	0.55
B2	41,408	864	1092.2	1129.3	4.04	0.946	0.60
C2	65,131	1090	1441.1	1479.3	3.42	0.895	0.49
D2	53,131	797	1114.5	1188.2	2.75	0.772	0.41
E2	29,441	1008	1315.5	1321.0	4.27	0.954	0.62
F2	73,023	978	1260.6	1281.1	3.58	0.926	0.52
G2	70,893	1013	1314.1	1300.2	3.30	0.875	0.48
H2	59,445	1090	1352.2	1339.2	3.68	0.904	0.53
I2	51,684	1122	1288.6	1279.8	4.56	0.966	0.65
J2	6975	714	925.0	911.0	4.80	0.964	0.73
Mean	47,636	947	1224.6	1209.4	3.81	0.908	0.56

## Data Availability

The data presented in this study are available on request from the corresponding author.

## Supplementary Materials

The following supporting information can be downloaded at: https://www.mdpi.com/article/10.3390/ijerph192113948/s1, Table S1: Descriptive statistics of the physicochemical properties at different sites before and after the floods; Table S2: Comparisons of the eukaryotic communities at different category levels before and after the floods; Table S3: The biotic dissimilarities of the spatial turnover and nestedness pattern indexes.
