# Supplementary material for: Changes in Microeukaryotic Communities in the Grand Canal of China in Response to Floods
Source: Int J Environ Res Public Health. 2022 Oct 27;19(21):13948. doi: 10.3390/ijerph192113948 (PMC9655333; doi:10.3390/ijerph192113948)
Supplement: Supplementary file 1 [file ijerph-19-13948-s001.zip › ijerph-1967998-supplementary.pdf]

Supporting Information

The calculations of Sørensen dissimilarity index (β_{SOR}), Simpson dissimilarity index (β_{SIM}), and nestedness-resultant dissimilarity index (β_{NES}) are as followed.

β_{SIM} depicts the spatial turnover of species among multipoint eukaryotes without the impact of richness gradients. It can be formulated as [21]:

$$\beta_{SIM} = \frac{\left[\sum_{i<j} \min(b_{ij}, b_{ji})\right]}{\left[\sum_{i} S_i - S_T\right] + \left[\sum_{i<j} \min(b_{ij}, b_{ji})\right]}$$

Where b_{ij} and b_{ji} are the number of species exclusive to sites i and j, respectively; $\left[\sum_{i<j} \min(b_{ij}, b_{ji})\right]$ is the multiple-site analogue of the matching components; S_i is the total specie number in site i; S_T is the total specie number in all sites together; $\left[\sum_{i} S_i - S_T\right]$ is the analogue of the a-component.

β_{SOR} is associated with the percentage of different species shared among various communities and is formulated as [21]:

$$\beta_{SOR} = \frac{\left[\sum_{i<j} \min(b_{ij}, b_{ji})\right] + \left[\sum_{i<j} \max(b_{ij}, b_{ji})\right]}{2\left[\sum_{i} S_i - S_T\right] + \left[\sum_{i<j} \min(b_{ij}, b_{ji})\right] + \left[\sum_{i<j} \max(b_{ij}, b_{ji})\right]}$$

Where $\left[\sum_{i<j} \max(b_{ij}, b_{ji})\right]$ is another multiple-site analogue of the matching components of pairwise measures.

β_{NES} indicates the species nestedness among multipoint biotas and can be aquired as [21]:

$$\beta_{NES} = \beta_{SOR} - \beta_{SIM} = \frac{\left[\sum_{i<j}\min(b_{ij},b_{ji})\right]+\left[\sum_{i<j}\max(b_{ij},b_{ji})\right]}{2\left[\sum_i S_i - S_T\right]+\left[\sum_{i<j}\min(b_{ij},b_{ji})\right]+\left[\sum_{i<j}\max(b_{ij},b_{ji})\right]} - \frac{\left[\sum_{i<j}\min(b_{ij},b_{ji})\right]}{\left[\sum_i S_i - S_T\right]+\left[\sum_{i<j}\min(b_{ij},b_{ji})\right]}$$

$$= \frac{\left[\sum_{i<j}\max(b_{ij},b_{ji})\right]-\left[\sum_{i<j}\min(b_{ij},b_{ji})\right]}{2\left[\sum_i S_i - S_T\right]+\left[\sum_{i<j}\min(b_{ij},b_{ji})\right]+\left[\sum_{i<j}\max(b_{ij},b_{ji})\right]} \times \frac{\sum_i S_i - S_T}{\left[\sum_i S_i - S_T\right]+\left[\sum_{i<j}\min(b_{ij},b_{ji})\right]}$$

Table S1 Descriptive statistics of the physicochemical properties at different sites before and after floods

Sample ID	T °C	pH	DO mg/L	Cond μs/cm−	COD_{Mn} mg/L	TN mg/L	TP mg/L
	Before Floods						
A1	19.3±0.1	7.86±0.31	6.1±1.1	408±27	3.53±0.24	3.28±0.15	0.84±0.10
B1	18.9±0.2	7.69±0.17	5.2±0.7	477±36	4.48±0.37	5.82±0.18	0.93±0.07
C1	19.1±0.1	8.03±0.14	5.3±0.9	412±18	5.32±0.18	4.33±0.31	1.21±0.04
D1	19.4±0.2	7.91±0.38	4.8±0.3	525±22	6.11±0.29	4.92±0.26	1.42±0.14
E1	18.8±0.1	8.05±0.12	6.2±0.4	508±26	4.19±0.41	5.39±0.21	1.68±0.17
F1	19.5±0.1	8.01±0.20	4.7±0.4	447±33	5.82±0.34	7.44±0.28	1.24±0.09
G1	19.2±0.3	7.85±0.17	3.3±0.3	482±17	6.62±0.36	6.02±0.42	1.02±0.13
H1	19.3±0.2	7.92±0.28	4.9±0.5	426±31	5.13±0.25	4.13±0.19	0.53±0.16
I1	19.3±0.1	7.78±0.24	6.3±0.2	389±22	4.25±0.17	5.21±0.24	1.08±0.18
J1	19.1±0.2	7.98±0.19	8.7±0.3	273±19	2.53±0.29	1.04±0.13	0.22±0.12
Mean	19.2±0.2	7.91±0.22	5.6±0.5	435±25	4.80±0.29	4.76±0.24	1.02±0.12
	After Floods						
A2	25.3±0.3	7.65±0.14	4.2±0.8	533±18	6.59±0.17	5.28±0.21	0.93±0.08
B2	25.7±0.1	7.71±0.18	4.8±0.5	587±31	5.78±0.23	5.03±0.15	1.18±0.11
C2	25.1±0.1	7.91±0.22	3.5±0.9	629±24	6.92±0.21	7.33±0.26	1.32±0.09
D2	25.4±0.2	7.74±0.17	3.9±0.3	548±26	7.87±0.26	4.29±0.28	1.19±0.21
E2	25.4±0.1	7.82±0.27	4.7±0.4	482±29	7.23±0.16	3.12±0.13	0.57±0.14
F2	25.6±0.2	7.90±0.24	5.3±0.7	411±15	6.71±0.18	4.35±0.17	0.42±0.18
G2	25.2±0.1	7.73±0.31	3.6±0.2	379±17	6.27±0.25	4.18±0.18	0.61±0.13
H2	25.1±0.3	7.81±0.26	2.8±0.5	473±22	7.82±0.29	6.23±0.14	0.94±0.09
I2	25.3±0.1	7.77±0.23	4.1±0.5	583±25	7.14±0.30	4.39±0.24	1.02±0.08
J2	25.1±0.2	7.62±0.16	4.6±0.3	502±16	6.53±0.27	5.28±0.18	1.33±0.18
Mean	25.3±0.2	7.77±0.22	4.2±0.5	513±22	6.89±0.23	4.95±0.19	0.95±0.13

Table S2 Comparisons of the eukaryotic communities at different category levels before and after floods

Category		Kingdom	Class	Order	Family	Genus
Groups	Group I (Microbial communities detected in at least 80% sites before flood, but not detected in at least 80% sites after flood)	-	*Pezizomycetes, Catenulida, Glomeromycetes, Ellipura*	*Pezizales, Tylenchida, Collembola*	*Rhizinaceae, Stenostomidae*	*Phymatotrichopsis, Gonostomum Hanseniaspora, Angulamoeba*
	Group II (Microbial communities detected in at least 80% sites both before and after flood)	*Fungi, Stramenopiles, Metazoa_Animalia, Chloroplastida, Alveolata, Discoba, Rhizaria, Amoebozoa, Cryptophyceae, Incertae_Sedis and Centrohelida. Stramenopiles, Metazoa_Animalia*	*Diatomea, Clitellata, Agaricomycetes, Maxillopoda, Kinetoplastea, Intramacronucleata, Embryophyta, Chlorophyceae, Cercomonadidae, Chrysophyceae, Incertae_Sedis, Trebouxiophyceae, Thecofilosea, Conoidasida, Bivalvia, Eurotiomycetes, Flabellinia, Chromadorea, Dothideomycetes, Tetramitia, Euamoebida, Euglenida, Eustigmatophyceae,*	*Metakinetoplastina, Bacillariophytina, Conthreep, Euglenophyceae, Prokinetoplastina, Spirotrichea, Litostomatea, Dactylopodida, Chaetonotida; Gregarinasina, Coscinodiscophytina, Saccharomycetales, Silicofilosea, Rhizophydiales, Incertae_Sedis, Ochromonadales, Chromulinales, Centramoebida,*	*Neobodonida, Mediophyceae, Bacillariophyceae, Amphitraemidae, Hypotrichia, Colpodea, Eugregarinorida, Oligohymenophorea, Haptoria, Incertae_Sedis, Melosirids, Trypanosomatida, Rotaliida, Euglyphida, Prostomatea, Phyllopharyngea, Choreotrichia, Nucleariidae,*	*Thalassiosira, Cyclotella, Melosira, Navicula, Ichthyobodo, Skeletonema, Neobodo, Cercomonas, Rhynchomonas, Pleurosigma, Nitzschia, Aulacoseira, Gymnophrys, Acanthamoeba, Sorodiplophrys, Korotnevella, Tintinnidium, BOLA868,*

			Longamoebia, Vampyrellidae, Phytomyxea, Arcellinida, Imbricatea, MAST-12C, Ulvophyceae, Leotiomycetes, Foraminifera, Xanthophyceae, Tremellomycetes, Aphelidea, Saccharomycetes	*Haplotaxida, Globothalamea, Liliopsida, Pleosporales, Eustigmatales, Armophorea, Sphaeropleales, Echinamoebida, Calanoida, Eurotiales, Mortierellales, Mytiloida, Rosales*	*Plagiopylea, Mortierellaceae, Aspergillaceae*	*Spumella, Aspergillus, Breviata, Mortierella, Ischnamoeba, Poteriospumella, Vermamoeba, Naegleria, Filamoeba Allovahlkampfia, Sellaphora, Woronina, Nuclearia*
	Group III (Microbial communities detected in at least 80% sites after flood, but not detected in at least 80% sites before flood)	-	-	*Diptera, Synurales, Hibberdiales*	*Pichiaceae, Oligotrichia, Armophorida*	*Acineta, Halteria, Epiphyllum, Brachonella, Paulinella, Mallomonas, Lepocinclis, Katablepharis, Pichia, Rhinosporidium*

Table S3 The biotic dissimilarities of the spatial turnover and nestedness pattern indexes

Group	β_{SOR}	β_{SIM}	β_{NES}
**Samples divided into 2 groups**			
BF (Sample A1, B1, C1, D1, E1, F1, G1,	0.8082	0.7054	0.1027
AF (Sample A2, B2, C2, D2, E2, F2, G2,	0.7318	0.6858	0.0459
Mean	0.7700	0.6956	0.0743
**Samples divided into 4 groups**			
BFS (Sample A1, B1, C1, D1 and E1)	0.6673	0.5813	0.0859
BFN (Sample F1, G1, H1, I1 and J1)	0.7275	0.6672	0.0603
AFS (Sample A2, B2, C2, D2 and E2)	0.6054	0.5520	0.0533
AFN (Sample F2, G2, H2, I2 and J2)	0.6002	0.5410	0.0592
Mean	0.6501	0.5854	0.0647
